# A Comparative Study on the Branching Pattern of Monocyclic and Bicyclic Shoots of Apple cv. “Fuji”

**DOI:** 10.3389/fpls.2020.571918

**Published:** 2020-08-31

**Authors:** Ying-Tsui Wang, Benoît Pallas, Melba R. Salazar-Gutierrez, Evelyne Costes, Gerrit Hoogenboom

**Affiliations:** ^1^AgWeatherNet Program, Washington State University, Prosser, WA, United States; ^2^Department of Biological Systems Engineering, Washington State University, Prosser, WA, United States; ^3^UMR AGAP, Univ Montpellier, CIRAD, INRAE, Institut Agro-Montpellier SupAgro, Montpellier, France

**Keywords:** hidden semi-Markov chain, rhythmicity, polycyclism, growth duration, growth period, *Malus* x *domestica*, tree architecture

## Abstract

The development of tree architecture results from shoot growth and branching, but their relationship is still not fully understood. The goal of this study was to determine the effect of parent shoot growth characteristics on branching patterns in terms of polycyclism, growth duration (GD), and growth period (GP), considering apple tree as a case study. Weekly shoot growth records were collected from 227 shoots during their second year of growth and the resulting branching patterns from the following year. The branching patterns were compared between the different shoot categories, using hidden semi-Markov models. Our results showed that the branching pattern was similar in bicyclic and monocyclic shoots with a long GD. The number of floral laterals, and the frequency and length of the floral zones, increased with GD. Moreover, a long GD led to strong acrotony, due to the high occurrence of a vegetative zone with long laterals in the distal position of the shoot. In bicyclic shoots, an early GP of the second GU led to more frequent and longer floral zones than a late GP. Therefore, the GD was the strongest driver of the branching pattern, and GP modulated the flowering capacity. The main similarities among shoot categories resulted from the existence of latent buds and floral zones associated with growth cessation periods. Even though flowering was more abundant during the early GP, the positions of floral zones indicated that induction in axillary meristems can also occur late in the season. This study provides new knowledge regarding the relationships between the dynamics of parent shoot growth and axillary meristem fates, with key consequences on flowering abundance and positions.

## Introduction

Tree architecture is described as a series of repetitive processes that build sequences of fundamental structural units called metamers (White, 1979; Barlow, 1994). A metamer consists of a node, a leaf, a lateral bud, and an internode. Depending upon the species, each lateral bud has the potential to either develop as a vegetative or reproductive shoot. This phenomenon together with the intrinsic ability of each bud to growth has been shown to generate patterns of lateral buds along parent shoots, which determine the tree canopy structure and affect plant production potential. Therefore, a better understanding of the crown architecture of trees could help fruit tree growers improve their orchard management strategies for training and pruning (Lauri, 2008; Lauri et al., 2011).

In most trees, different shoot types are classified according to their length and growth rhythm, with wide variations in the timing of growth cessation (Hallé et al., 1978; Crabbé, 1984). Shoots can cease growth early, after developing preformed organs that were initiated during the previous season; or they can cease growth later, when neoformed organs develop after the preformed organs. With sufficient resources, shoots can keep growing after the extension of the preformed organs. In this case, new (neoformed) metamers are produced regularly by the apical meristem at the shoot tip until a rest period. The neoformation capability and duration have been described as a key contribution to the shoot plasticity within a tree (Davidson and Remphrey, 1994). In addition, shoots can display polycyclic growth, which is defined as more than one growth cycle during the same growing season (Hallé et al., 1978). A shoot portion that develops during an uninterrupted growth cycle is called a growth unit (GU). This periodic shoot extension can be identified by the presence of bud scars or very short internodes between the GUs that developed in the same year (deReffye et al., 1991; Barthélémy and Caraglio, 2007).

Although shoot growth and branching patterns have been studied for many species, including fruit trees (Costes and Guédon, 2002; Renton et al., 2006; Negrón et al., 2013; Prats-Llinàs et al., 2019), their relationship is still not fully understood. Many studies have shown that the development of different types of lateral buds is related to their within-shoot location (Lauri and Terouanne, 1998; Guédon et al., 2001; Costes and Guédon, 2002). In trees, usually the most distal lateral buds develop into long shoots, with a decrease in length from the top of the bearing shoots, a phenomenon known as acrotonic gradient (Cook et al., 1998). In apple trees (*Malus* x *domestica*), floral buds are located below the acrotonic zones (Crabbé, 1984; Lauri and Terouanne, 1998). The floral bud develops into a short swollen axis, called bourse, with rosette leaves at the basal part and an inflorescence at the terminal part (Pratt, 1988), and, therefore, corresponds to a mixed inflorescence. The bourse may bear one or two vegetative shoots, called bourse shoots, that developed from axillary buds on the bourse. Sylleptic branching, which results from a rapid growth rate, may appear in the median position of long parent shoots (Crabbé, 1984; Costes and Guédon, 1997; Costes and Guédon, 2002).

To quantify branching patterns, a statistical modeling approach has been proposed for several fruit species, such as apple (Costes and Guédon, 1997; Costes and Guédon, 2002; Renton et al., 2006), peach (Fournier et al., 1998; Prats-Llinàs et al., 2019), apricot (Costes and Guédon, 1996), *Actinidia* (Seleznyova et al., 2002), and almond (Negrón et al., 2013). These species have a zonation of different lateral bud fates that can be modeled with hidden semi-Markov chains (Guédon et al., 2001; Guédon, 2003). These statistical models are suitable for identifying successions in discrete sequences of zones in which composition properties are homogeneous within the zones but variable between zones. Costes and Guédon (1997; 2002) were the first to use the hidden semi-Markov chains to analyze the distribution of sylleptic and proleptic shoots along the trunks of 1-year-old trees for six apple cultivars. Four common zones, including the basal latent zone, middle sylleptic zones, floral zones, and acrotonic zones, were recognized across the cultivars and presented in a similar order along the parent shoots. The hidden semi-Markov chains have also been used for investigating the similarities and morphogenetic gradients of branching patterns along GUs of apple trees with different lengths (Renton et al., 2006). The decrease in length of the parent GUs during tree ontogeny led to a progressive simplification of branching patterns and the reduction in the length of the floral zones. The probability of occurrence of the floral zone also varied with years, probably due to alternate bearing in this species. However, the position of the flower zone remained unchanged and was consistently located in the top third of the GU.

A relatively high proportion of polycyclic growth of annual shoots has been observed in young apple trees (Seleznyova et al., 2008; Costes and Guédon, 2012). As trees age, shoot growth and polycyclism decline in many cultivars (Costes et al., 2003) and rootstocks (Seleznyova et al., 2003; Seleznyova et al., 2008). It has been reported that polycyclic shoots have distinctive growth features between GUs (Barthélémy and Caraglio, 2007). Lauri and Terouanne (1998) have shown that the two GUs of bicyclic annual shoots of apple trees present different morphometric characteristics and axillary organogenetic activities. The first GU has large variations in internode length and leaf size and was characterized by a low axillary organogenetic activity, whereas the second GU is characterized by stronger axillary organogenetic activity.

To date, studies of branching patterns of apple trees have mainly focused on annual shoots that are collected from unpruned trees, with growth starting in the spring. However, so far no research has been conducted to investigate the changes of branching patterns of GUs with respect to different growth periods, i.e., time of budbreak, and growth durations, i.e., the length of time from budbreak to cessation. The goal of this study was to determine the effect of shoot growth characteristics in terms of duration, rhythmicity, and period of growth on the branching patterns using hidden semi-Markov chains. Our hypothesis was that similarities could be identified along the shoots, with either repeated and/or similar zones observed based on either the growth duration or period. The similarities and differences of branching patterns were, therefore, analyzed among shoot and GU categories.

## Materials and Methods

### Plant Material

The research was conducted in a high-density commercial apple orchard, with about 4500 trees/ha, located in Prosser, Washington, in the United States. The apple tree cultivar “Fuji” was grafted on rootstock “Nic29” and planted during the spring of 2015. Trees were cultivated under standard irrigation and fertilization conditions recommended by the Washington State University (WSU) Tree Fruit Research and Extension (http://treefruit.wsu.edu/). The apical shoots and long lateral shoots along the trunks were removed immediately following planting. Two pruning strategies were applied in the following years on two sets of five trees each. In the first set, selected long lateral branches were thinned back to the trunk in the winter of both 2015 to 2016 and 2016 to 2017, with a pruning strategy that followed the V-trellis planting system (Robinson, 2003). In addition, long laterals on the main stem of trunks were removed in the summer on May 25, 2016. In the second set of trees, some lateral branches along the trunks were partially pruned during the summer on May 25, 2016 and no additional winter pruning was applied. The long laterals on the main stem of trunks were removed in the summer on May 25, 2016. More severe summer pruning of lateral branches was conducted at the top of the trunks than at the bottom to decrease competition with the growth of the trunks. All fruits were removed in mid-May in both experiments. In this study, additional details with respect to pruning strategies such as the number and proportion of removed shoots are not described because we did not aim to investigate the differences in branching patterns between two sets of trees. The analysis of branching patterns focused on the effects of polycyclism, growth period, and duration, which in turn are influenced by pruning.

The length of all shoots was measured weekly in 2016 for ten trees. At the end of the 2017 growing season, the branching patterns were recorded for (1) unpruned shoots that had grown throughout the entire 2016 growing season and (2) new shoots that had grown after summer pruning. The shoots that were pruned in winter and the segments of summer pruned shoots that grew before pruning were not considered. The branching patterns were described by sequences of symbols representing the fate of each lateral bud borne on a node from the base to the top of each shoot. Similar to previous studies (Costes and Guédon, 2002; Costes et al., 2003; Renton et al., 2006), five types of lateral buds were considered: 0 for a latent bud, 1 for a short shoot (< 5 cm), 2 for a long shoot (≥ 5 cm), 3 for a bourse with a short bourse shoot, and 4 for a bourse with a long bourse shoot. Here, a bourse with no bourse shoot was included in the category of a bourse with short bourse shoot. If two bourse shoots were observed, only the longer one was recorded for branching pattern analysis. A bourse with bourse shoot that developed from a lateral floral bud was referred to as floral lateral. The number of flowers and fruits per bourse were not investigated in this study. As a consequence all the floral laterals were considered as belonging to the same class in the following analysis whatever the number of flowers per inflorescence.

### Grouping the Sequences Based on Growth Cessation, Duration, and Period

**Figure 1:**
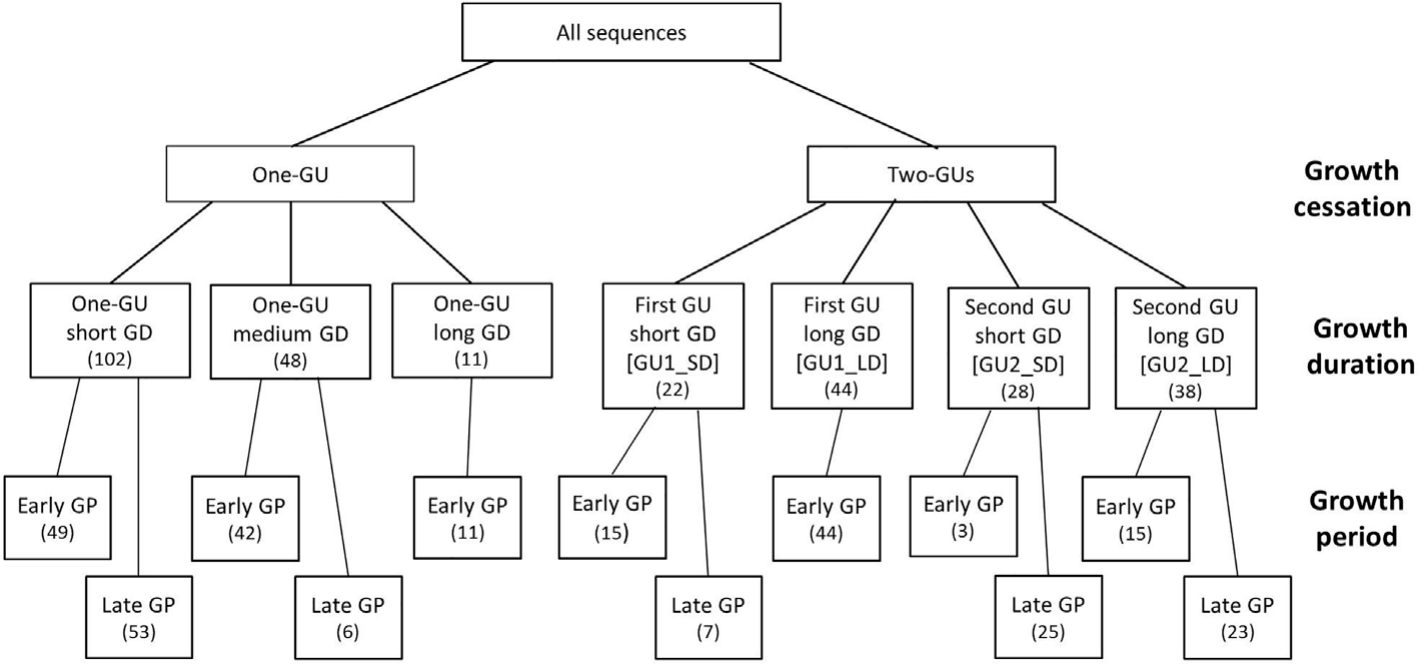
Diagrammatic representation of shoot categories based on the existence of within-year growth cessation, growth duration (GD), and growth period (GP). The category with one-GU shoots was divided by GD thresholds of 30 and 90 days, and a GP threshold of June 3, 2016. Both GUs of two-GUs shoots were divided by a GD threshold of 30 days. Thresholds of GP were June 3, 2016 for the one-GU shoots and the first GUs, and July 18, 2016 for the second GUs. The number of sequences for each category is in the bracket.

A total of 227 shoots were observed from the ten trees and were classified according to their growth characteristics, i.e., the existence of a within-season cessation, growth durations, and periods (**Figure 1**). Growth cessations were identified when the increase in shoot length was less than 0.5 cm over two weeks. Growth durations (GD) were calculated as the difference between the starting and cessation dates. The starting date was defined as the date when shoots reached 5 cm. The mean starting date occurred on April 30, 2016 in spring for shoots growing before summer pruning. Late growth was also observed for the new shoots released after the summer pruning, and their mean starting date was on June 26, 2016.

Based on the existence of within-season growth cessation, all shoots were separated into two groups, i.e., one-GU and two-GUs. The within-season cessation was identified by the presence of bud scars. Each group was further divided according to the growth duration (GD). The one-GU shoots contained three subgroups, short (< 30 days), medium (30–90 days), and long (> 90 days) GD, respectively. For the two-GU shoots, each GU was considered separately. The first and second GUs were divided into two subgroups, corresponding to short or long GD with respect to a threshold of 30 days. Next, each subgroup was divided into early and late growth period (GP), based on the starting date threshold of June 3, 2016 for the one-GU shoots and the first GUs of the two-GU shoots and the threshold of July 18, 2016 for the second GUs.

### Node Number and Number of Lateral Types

The statistical analyses on total node number, number of different lateral types, and lateral-type distribution per shoot and GU, were conducted among different categories grouped by GD and GP. Generalized linear models (GLMs) and non-parametric tests were used on count data and when the data did not follow normal distributions. Using GLM, the goodness-of-fit of Poisson or negative binomial distribution was tested with a chi-square test before selecting the model.

The comparisons were first conducted among the four shoot categories, including one-GU with short, medium and long GD, and two-GUs. The node number of shoots was compared among four shoot categories using the Poisson GLM followed by a post-hoc Tukey test. The number and proportion of laterals per shoot, including latent buds, short shoots, long shoots, short bourse shoots, and long bourse shoots, were compared using the Kruskal-Wallis test followed by post-hoc Dunn test, and chi-squared test followed by post-hoc Tukey test, respectively. The Kruskal-Wallis test was used when comparing the differences among more than two groups. The similar comparisons were conducted for each GU of the two-GU shoots between the two GD subgroups, i.e. first GU short GD vs. long GD and second GU short GU vs. long GD, and between two GP subgroups, i.e., early vs. late GP. The Mann-Whitney test was used for comparison of the number of laterals per shoot/GU, and Fisher’s exact test was for lateral-type distribution per shoot/GU. The Mann-Whitney test was used when comparing the differences between two groups. The Fisher’s exact test was used when the sample size was small for some cells of contingency tables.

### Estimation of Hidden Semi-Markov Chains for Branching Pattern Analysis

The fates of buds according to their within-shoot position were analyzed with hidden semi-Markov chains. We first estimated a single hidden semi-Markov chain considering all shoot categories together. However, a poor segmentation was obtained because of differing branching pattern, particularly in the distal part of the shoots for the shoots with different lengths or GDs.

We distinguished two sets of shoots that differed significantly in their total number of nodes: one-GU shoots with short and medium GD, and the one-GU long GD and two-GU shoots. Two hidden semi-Markov chain models were estimated for these two sets of shoots. The models contained successive states followed by a final absorbing state (see Guédon et al., 2001 and Guédon, 2003 for further details). Each model consisted of four sets of parameters: 1) initial probabilities that determine the first zone present at the base of the shoots, 2) transition probabilities between zones, 3) occupancy distributions representing the length of each zone in terms of node number, and 4) observation distributions representing the mixture of lateral bud fates within each zone (see Guédon et al., 2001 and Renton et al., 2006 for details). Both models were unidirectional, i.e., the transition was only allowed from left to right, and transient, i.e., impossible to stay in the states already visited. Additionally, the first states of both models were permitted to only have latent buds that correspond to the basal unbranched zone (Lauri and Terouanne, 1998; Costes and Guédon, 2002; Renton et al., 2006). The same restriction was applied for the first state of the second GU where a latent zone was also observed by Lauri and Terouanne (1998). The estimation of the hidden semi-Markov chain models was conducted using the VPlants software, which is part of the OpenAlea platform (Pradal et al., 2008).

### Comparison of Model Parameters According to Growth Cessation, GD, and GP

Once the models were estimated, the most probable state sequences that corresponded to the optimal segmentation of the observed sequences into branching zones were extracted for each shoot. The probability of occurrence of each state and the transition probability between each state were analyzed for the different shoot and GU categories grouped by the existence of growth cessation, GD, and GP. The zone lengths were also compared using a negative binomial GLM, and lateral-type distributions within the states were compared using Fisher’s exact test (P < 0.05). In addition, the relation between total shoot length and zone length was tested with a Pearson correlation analysis. All statistical analyses were performed using R version 3.4.1 (R Core Team, 2017).

## Results

### Number of Shoots per Growth Duration and Period

**Figure 2:**
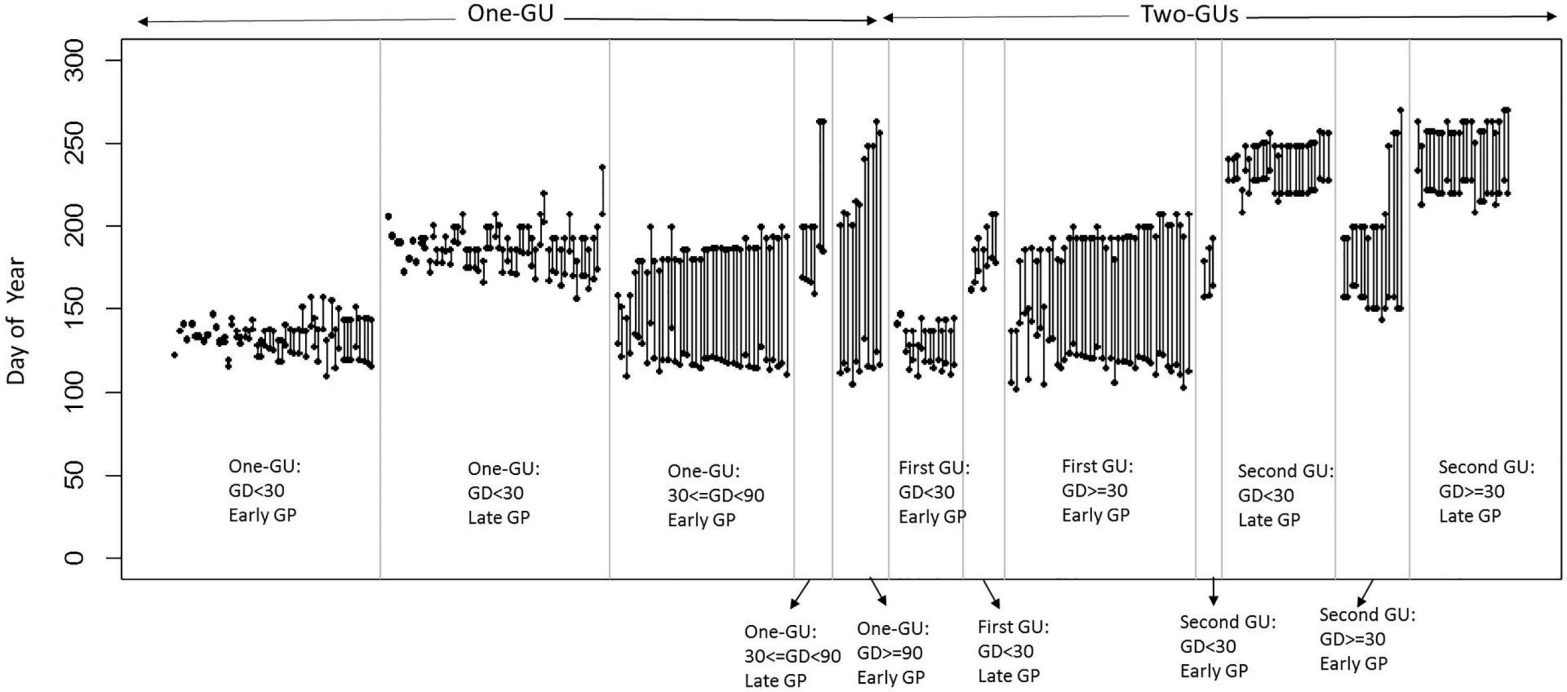
Growth duration (GD) and growth period (GP) of the GUs for the different shoot categories (one-GU and two-GUs). The dots represent the start and cessation dates of growth for each GU. Two GPs (early and late) are defined by a threshold of June 3, 2016, for one-GU and first GUs of two-GUs, and July 18, 2016, for the second GUs.

The sample of shoots consisted of 71% one-GU and 29% two-GUs (**Figure 2**). Among the one-GU shoots, a high proportion (65%) had a short GD and half grew during the late GP, after June 3, 2016. About 28% of the one-GU shoots had medium GD, 15% of them with a late GP. Only 7% of the one-GU shoots had long GD and all of them started to grow early (before June 3, 2016). For the two-GUs, a high proportion (67%) of the first GUs had a long GD and about 33% of the first GUs had a short GD. All of the first GUs with a long GD grew early, whereas for the first GU with short GD, only 68% of them grew early. The second GUs consisted of 42% with a short GD and 58% with a long GD. Most of the second GU with a short GD grew during the late GP (after July 18, 2016), while only three shoots grew early (before July 18, 2016) and were not considered in further analyses. More second GUs with a long GD grew late (61%) than early (39%).

### Total Node Number and Number of Lateral Types per Shoot

**Figure 3:**
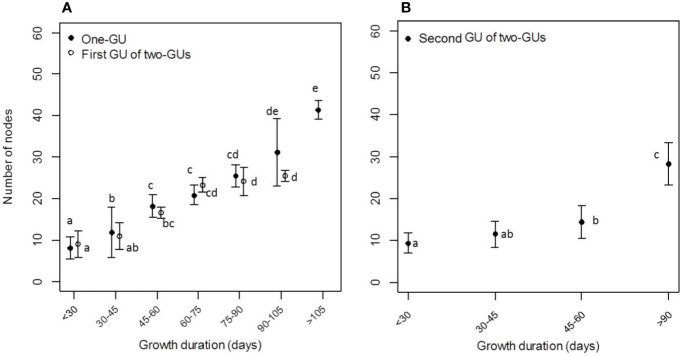
Mean node number and shoot length of the one-GU shoots **(A)**, first GU **(A)**, and second GU **(B)** of the two-GU shoots for different ranges of growth duration (GD). Different letters indicate significant differences in the number of nodes between different classes of GD for a given GU.

The mean number of nodes per shoot and GU increased with the GD, whereas the GP had a lower impact on node number when the comparisons were conducted between the early and late GP within different shoot/GU categories (**Figure 3**; **Table 1**). A significant impact of GP was only observed for the one-GU shoots with a short GD. There was an average of eight nodes per shoot on the one-GU shoots with a short GD, increasing up to 20 when the GD was medium (30–90 days). When the GD was long (> 90 days), the mean number of nodes per shoot also increased with growth duration, with an average of 35 nodes (**Figure 3A**; **Table 1**). The one-GU shoots had a similar node number with the first GUs of the two-GU shoots when the GD was less than 90 days (P > 0.05, Poisson GLM; **Figure 3A**). However, the one-GU shoots had more nodes than the first GUs of the two-GU shoots when the GD was long (> 90 days). A higher number of nodes was also observed for the one-GU shoots compared to the second GUs of the two-GU shoots when the GD was longer than 45 days (P < 0.05, Poisson GLM; **Figure 3A**).

**Table 1:** Mean number of nodes per parent shoot and growth unit (GU), and mean number and proportion (in parentheses) of lateral types (lateral buds, short shoots, long shoots, short and long bourse shoots) among categories grouped by growth cessation, growth duration (GD), and growth period.

Shoot categories^1^	Growth period^2^	Number of laterals					Total number of nodes
		latentbuds	Vegetative shoots		Bourse shoots		
			short	long	short	long	
One-GU (N = 161)							
Short GD	All	7.35^a^ (0.91)	0.19^a^ (0.02)	0.05^a^ (0.01)	0.46^a^ (0.06)	0.03^a^ (0.00)	8.09^a^ (a)
Medium GD	All	14.67^b^ (0.75)	0.46^a^ (0.02)	0.46^b^ (0.02)	2.96^b^ (0.15)	0.94^b^ (0.05)	19.48^b^ (b)
Long GD	All	28.18^c^ (0.79)	2.09^b^ (0.06)	1.27^c^ (0.04)	3.09^b^ (0.09)	1.18^b^ (0.03)	35.82^c^ (c)
Two-GUs (N = 66)	All	22.56^c^ (0.77)	2.03^b^ (0.07)	1.11^c^ (0.04)	3.15^b^ (0.11)	0.39^c^ (0.01)	29.24^c^ (c)
Two-GUs: First GU (N = 66)							
Short GD	All	8.23^a^ (0.91)	0.00^ns^ (0.00)	0.00^ns^ (0.00)	0.77^a^ (0.09)	0.00^a^ (0.00)	9.00^a^ (a)
Long GD	All	18.84^b^ (0.87)	0.14^ns^ (0.01)	0.00^ns^ (0.00)	2.36^b^ (0.11)	0.27^b^ (0.01)	21.61^b^ (a)
Two-GUs: Second GU (N = 66)							
Short GD	All	6.29^ns^ (0.68)	1.57^ns^ (0.15)	0.82^ns^ (0.09)	0.64^a^ (0.06)	0.07^ns^ (0.01)	9.39^a^ (a)
Long GD	All	7.97^ns^ (0.58)	2.21^ns^ (0.16)	1.32^ns^ (0.10)	1.82^b^ (0.13)	0.32^ns^ (0.02)	13.63^b^ (b)
One-GU							
Short GD	Early	6.14^a^ (0.91)	0.08^a^ (0.01)	0.00^ns^ (0.00)	0.49^ns^ (0.07)	0.02^ns^ (0.00)	6.73^a^ (a)
	Late	8.47^b^ (0.91)	0.30^b^ (0.03)	0.09^ns^ (0.01)	0.43^ns^ (0.05)	0.04^ns^ (0.00)	9.34^b^ (b)
Medium GD	Early	14.71^ns^ (0.76)	0.38^ns^ (0.02)	0.26^a^ (0.01)	3.14^ns^ (0.16)	0.90^ns^ (0.05)	19.40^ns^ (a)
	Late	14.33^ns^ (0.72)	1.00^ns^ (0.05)	1.83^b^ (0.09)	1.67^ns^ (0.08)	1.17^ns^ (0.06)	20.00^ns^ (b)
Two-GUs: First GU							
Short GD	Early	8.07 ^ns^ (0.90)	0.00^ns^ (0.00)	0.00^ns^ (0.00)	0.93^ns^ (0.10)	0.00^ns^ (0.00)	9.00^ns^ (a)
	Late	8.57 ^ns^ (0.95)	0.00^ns^ (0.00)	0.00^ns^ (0.00)	0.43^ns^ (0.05)	0.00^ns^ (0.00)	9.00^ns^ (a)
Two-GUs: Second GU							
Long GD	Early	10.93^ns^ (0.72)	0.73^a^ (0.05)	0.47^a^ (0.03)	2.87^a^ (0.19)	0.27^ns^ (0.02)	15.27^ns^ (a)
	Late	6.04^ns^ (0.48)	3.17^b^ (0.25)	1.87^b^ (0.07)	1.13^b^ (0.09)	0.35^ns^ (0.03)	12.57^ns^ (b)

^1^For the comparison of the lateral number and proportion (in parentheses) across four shoot categories (one-GU short GD, one-GU medium GD, one-GU long GD, two-GUs), different letters per column indicate significant differences (P < 0.05) according to Kruskal-Wallis test with post-hoc Dunn test and chi-squared test with post-hoc Tukey test, respectively. For the comparison of the lateral number and proportion between different GDs within the first and second GUs, different letters indicate significant differences (P < 0.05) according to the Mann-Whitney test and Fisher’s exact test, respectively. ^2^For the comparison of the lateral number and proportion between early and late growth period, different letters indicate significant differences (P < 0.05) according to the Mann-Whitney test and Fisher’s exact test, respectively. Ns indicates the test is non-significant (P ≥ 0.05).

A high proportion of latent buds was observed for all shoot categories (**Table 1**). For the one-GU shoots, the number of lateral vegetative and bourse shoots increased with the GD, but this increase was not directly associated with an increase in the proportion of these shoots. The two-GU shoots had a similar proportion of lateral types as the one-GU shoots with long GD. For the first GUs of the two-GU shoots, the numbers of latent bud and bourse shoot increased with GD, and the number of vegetative shoots remained close to 0 for both GD categories. The higher number of bourse shoots did not seem to be associated with higher proportions of bourse shoots for the first GUs. The second GUs had a higher number of short bourse shoots with a long GD than with a short GD, and this increase was associated with an increase in the proportion of lateral shoots, 13% vs. 6% for the short bourse shoots. However, the differences in the number of vegetative and bourse shoots did not exceed two per shoot.

Higher numbers of both short and long vegetative laterals were observed for all shoot and GU categories with a late GP compared to an early GP, except for the one-GU shoots with a short GD and the first GU with a short GD (**Table 1**). However, these differences in the number of vegetative laterals were less than three nodes. The higher number of vegetative laterals was associated with a higher proportion of vegetative laterals for the late GP compared to the early GP. The most noticeable difference was in the second GUs with a long GD that had a higher proportion of short vegetative laterals for the late GP than the early GP, i.e., 25% vs. 5%.

### General Models

**Figure 4:**
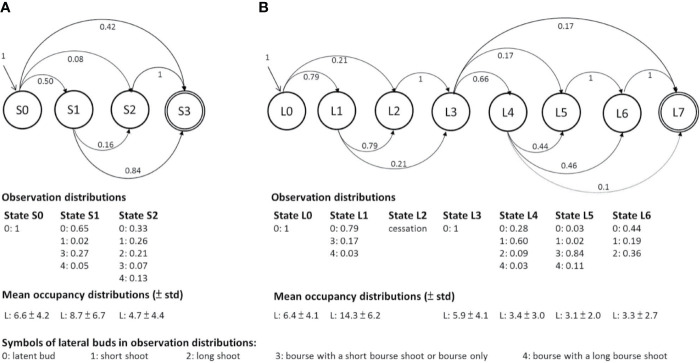
Graphic representation of the two hidden semi-Markov chain models estimated on **(A)** the one-GU shoots with short or medium growth duration (GD) and **(B)** the one-GU shoots with long GD and two-GU shoots. The branching zones are modeled by transient states, represented by circles with a single line border while the final absorbing states are represented by a double-line circle. Each state is associated with an observation distribution representing the specific mixture of lateral types in a zone and an occupancy distribution representing the mean number of nodes of each zone. The leftmost state (state S0 and L0) is the initial zone at the base of a shoot. The transition from one zone to another is represented by an arrow, with its probability indicated nearby. Only transition probabilities greater than 0.01 are shown.

The hidden semi-Markov chains for the one-GU with a short and medium GD included three transient states (S0, S1, S2) and an absorbing end state (S3) (**Figure 4A**). Each state was defined by its observation distribution and named for distinguishing lateral bud type population: 1) State S0 at the basal part of shoots consisted of latent buds only (hereafter referred to as basal latent zone); 2) State S1 was composed of a mixture of floral laterals mainly with short bourse shoot and latent buds (diffuse floral zone); 3) State S2 at the distal part of shoots consisted of short and long vegetative laterals mixed with latent buds and a few floral laterals with either short or long bourse shoots (acrotonic vegetative zone).

The hidden semi-Markov model for the one-GU with a long GD and two-GU shoots was composed of seven transient states (L0 to L6) and an absorbing end state (L7) (**Figure 4B**). The states were defined as follows: 1) State L0 was similar to S0 in the model for the one-GU with short and medium GD (basal latent zone); 2) State L1 consisted of a mixture of numerous latent buds and few floral laterals with a majority of shot bourse shoots (diffuse floral zone); 3) State L2 represented the bud scars or a succession of short internodes and was only observed in the two-GU shoots (cessation zone). The states before cessation zone were regarded as the first GU, and the states after were in the second GU; 4) State L3 consisted of latent buds only, and was located at the beginning of the second GU (latent zone); 5) State L4 was comprised by a mixture of short shoots and latent buds with few long vegetative shoots and floral laterals with long bourse shoots (short shoot zone); 6) State L5 consisted of a majority of floral laterals with short bourse shoots (floral zone); 7) State L6, located at the distal part of the shoots, was observed to contain a mixture of latent buds and vegetative shoots, with a majority of long lateral shoots (acrotonic vegetative zone).

### Branching Patterns for the One-GU and Two-GU Shoots

#### Effect of Growth Cessation on Branching Patterns

**Figure 5:**
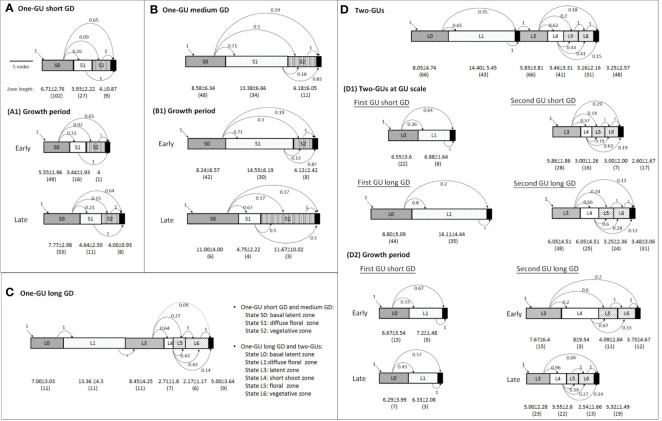
Schematic representation of branching patterns in four shoot categories **(A–D)**, subgroups of two-GU shoots (D1), and subgroups of the growth period (A1, B1, D2). The successive zones of the shoots are represented by rectangles. The number in the middle of the rectangles indicates the state numbers, as defined in the corresponding general model. The black rectangles at the right represent the absorbing end states while those located between states 1 and 3 in the two-GU shoots represent the growth cessation during a growing season. The transitions between states are indicated by arrows, with the associated probabilities indicated nearby. The number of zone (in brackets) and zone lengths for the number of metamers are indicated below each rectangle.

The one-GU shoots with short and medium GD had a simpler branching structure than the two-GU shoots (**Figures 4** and **5**). However, the branching patterns of each GU of the two-GU shoots and one-GU shoots with a short and medium GD showed similarities in the number and order of the states. The first GUs of two-GU shoots were quite similar to the one-GU shoots except that the acrotonic zone was not present in the first GU of the two-GU shoots (**Figure 5**; **Table 1**). In the second GU of two-GU shoots, the vegetative and floral zones were separated, i.e., one zone for vegetative laterals (L6) and one zone for floral laterals (L5), whereas the two types of laterals were observed in a single zone, i.e., S2, in the one-GU shoots with short and medium GD.

**Figure 6:**
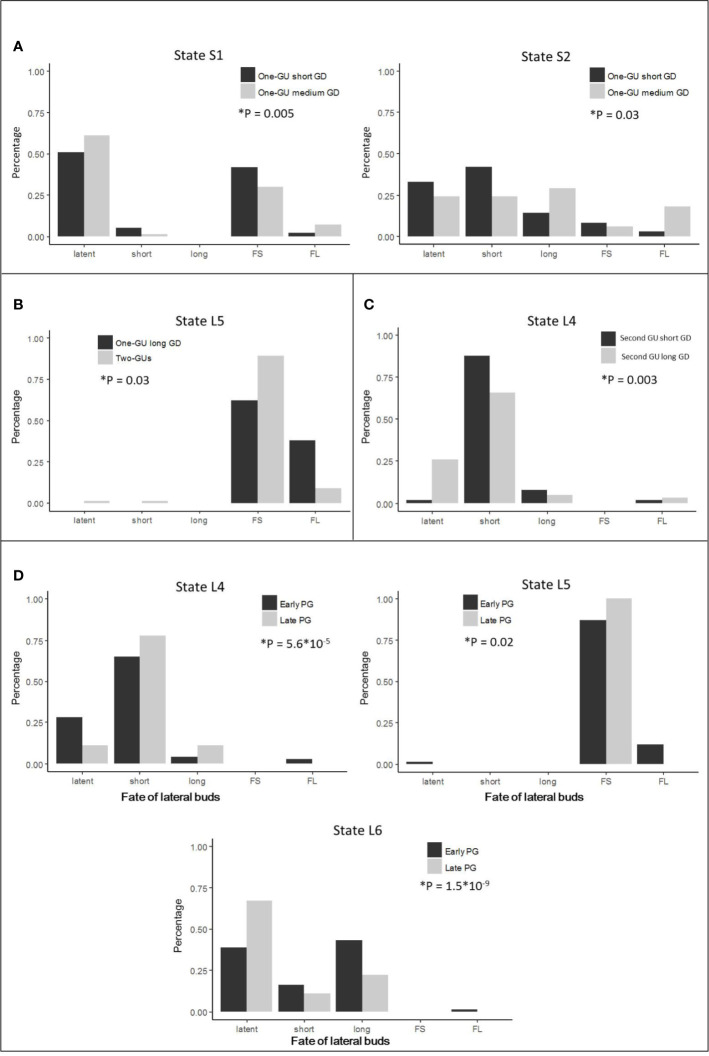
The distribution of lateral types per state, extracted from the most probable state sequences estimated by the general hidden semi-Markov models for different parent shoot categories. The lateral types are latent buds (latent), short shoots (short), long shoots (long), floral units with a short bourse-shoot (FS), and floral units with a long bourse-shoot (FL). The distributions are compared for each state with more than one lateral type between **(A)** one-GU shoots with short versus medium growth duration (GD), **(B)** one-GU shoots with long GD versus two-GUs shoots, **(C)** second GU of two-GU shoots with short versus long GD **(D)** second GU long GD with an early versus a late growth period (GP). The comparison was performed using Fisher’s exact tests. The test is significant (*) when P <0.05.

The one-GU shoots with a long GD showed great similarities with the two-GU shoots in the number and length of zones (**Figures 5C, D**; **Table 3**). A latent zone can also be observed in the middle portion of the one-GU shoots with a long GD. Nevertheless, minor differences were found in the occurrence probability of zones and the lateral-type distribution of floral zone (L5) (**Table 2**; **Figure 6B**). The diffuse flower zone (L1) was present in all one-GU shoots with a long GD while it could be skipped in the two-GU shoots (65%). The other zones (L4, L5, and L6) had a very similar probability of occurrence in the one-GU shoots with a long GD and the two-GU shoots, despite being lightly lower for L5 and L6 in the two-GUs. The floral zone (L5) had a higher proportion of floral laterals with long bourse shoots and a lower proportion with short bourse shoots for the one-GU with a long GD than the two-GU shoots (**Figure 6B**). No significant differences in the lateral-type distribution were found for the other zones (L1, L4, and L6; data not shown).

**Table 2:** Occurrence probability of each branching zone for shoots grouped by growth cessation, growth duration (GD), and growth period.

Shoot categories		Growth period	States					
A. Model for one-GU short and medium GD			S0	S1	S2			
One-GU short GD		All	1	0.26	0.09			
One-GU medium GD		All	1	0.71	0.23			
One-GU short GD		Early	1	0.33	0.02			
		Late	1	0.21	0.15			
One-GU medium GD		Early	1	0.71	0.19			
		Late	1	0.67	0.50			
B. Model for one-GU long GD and two-GUs			L0	L1	L3	L4	L5	L6
One-GU long GD		All	1	1	1	0.64	0.55	0.82
Two-GUs		All	1	0.65	1	0.62	0.47	0.73
	Subgroups^1^							
Two-GUs	GU1_SD	All	1	0.36	─	─	─	─
	GU1_LD	All	1	0.80	─	─	─	─
	GU2_SD	All	─	─	1	0.57	0.25	0.61
	GU2_LD	All	─	─	1	0.66	0.63	0.82
Two-GUs	GU1_SD	Early	1	0.33	─	─	─	─
		Late	1	0.43	─	─	─	─
	GU2_LD	Early	─	─	1	0.20	0.73	0.80
		Late	─	─	1	0.96	0.57	0.83

^1^GU1_SD and GU1_LD are the first GU with short and long growth duration, respectively; GU2_SD and GU2_LD are the second GU with short and long growth duration, respectively.

**Table 3:** Statistical comparison of zone length among shoot categories grouped by growth cessation, growth duration (GD), and growth period (GP).

Comparisons	States					
A. Model for one-GU short and medium GD	S0	S1	S2			
One-GU: short vs. medium GD	*	**	*			
One-GU short GD: early vs. late GP	**	ns	─			
One-GU medium GD: early vs. late GP	ns	─	─			
B. Model for one-GU long GD and two-GUs	L0	L1	L3	L4	L5	L6
One-GU long GD vs. two-GUs	ns	ns	ns	ns	ns	Ns
Two-GUs: GU1_SD vs. GU1_LD	ns	**	─	─	─	─
Two-GUs: GU2_SD vs. GU2_LD	─	─	ns	ns	*	Ns
GU1_SD: early vs. late GP	ns	─	─	─	─	─
GU2_LD: early vs. late GP	─	─	ns	─	ns	Ns

Significant differences were tested by GLM based on negative binomial distributions (** when P is <0.01 and * when P is 0.01–0.05). Ns indicates that the test is non-significant (P ≥ 0.05). Tests were performed for N > 5 only.

#### Effect of Growth Duration on Branching Patterns

The one-GU shoots with either a short or medium GD exhibited a similar structure, with zones in the same relative position along shoots. Their branching pattern, however, differed in zone occurrences (**Table 2**; **Figure 5**), zone lengths (**Table 3**; **Figure 5**), and lateral-type distribution within zones (**Figure 6A**). First, for the shoots with a short GD, the median zones, i.e., the diffuse floral zone (S1) and the acrotonic vegetative zone (S2), were often skipped leading to unbranched shoots (65%). Only 35% of shoots contained a branched zone, either a diffuse floral zone (26%) or a vegetative zone (9%), and none of the shoots exhibited both zones. In contrast, most of the one-GUs with a medium GD contained one branched zone, either a diffuse floral zone (59%) or a vegetative zone (13%) (**Figure 5B**; **Table 3**). About 13% of the shoots contained both zones, and only 15% were unbranched. The mean zone lengths also increased with the GD, especially the diffuse floral zone (S1), which was three times longer for the one-GU with a medium GD than the short GD, 13.4, and 3.9 nodes, respectively (**Figure 5**; **Table 3**). As a result, the one-GU shoots with a medium GD contained more floral laterals, having more frequent and longer floral zones than shoots with a short GD. Also, the one-GU shoots with a short GD had a lower proportion of long laterals (either vegetative or bourse shoot) compared to the one-GU shoots with a medium GD in the diffuse floral (S1) and vegetative zones (S2) (**Figure 6A**). Since S2 was rarely observed in the one-GU shoots with a short GD, acrotony remained more pronounced in the one-GU shoots with medium GD, displaying more numerous long laterals in the distal portion.

Similarly, GD affected the branching patterns of each GU in the two-GU shoots. Differences were found in the zone occurrence, zone length, and lateral-type distribution. The first GU had a higher tendency to develop the diffuse floral zone (L1) when the GD was long compared to when it was short (**Table 2**). The same pattern was also observed for the floral zone (L5) in the second GU. The mean length of the floral zones (L1 and L5) was significantly longer for both first and second GUs with a long GD (16.1 and 3.3 nodes, respectively) compared to those with a short GD (6.9 and 2.4 nodes, respectively) (**Figure 5 D1**; **Table 3**). The GD did not have a significant effect on the length of other zones for either GUs of the two-GU shoots (state L0, L3, L4, and L6). Additionally, a slight difference of lateral-type distribution was observed in the short shoot zone (L4) with a higher proportion of short laterals and lower proportion of latent buds in the GUs with a short GD than with a long GD (**Figure 6C**).

**Figure 7:**
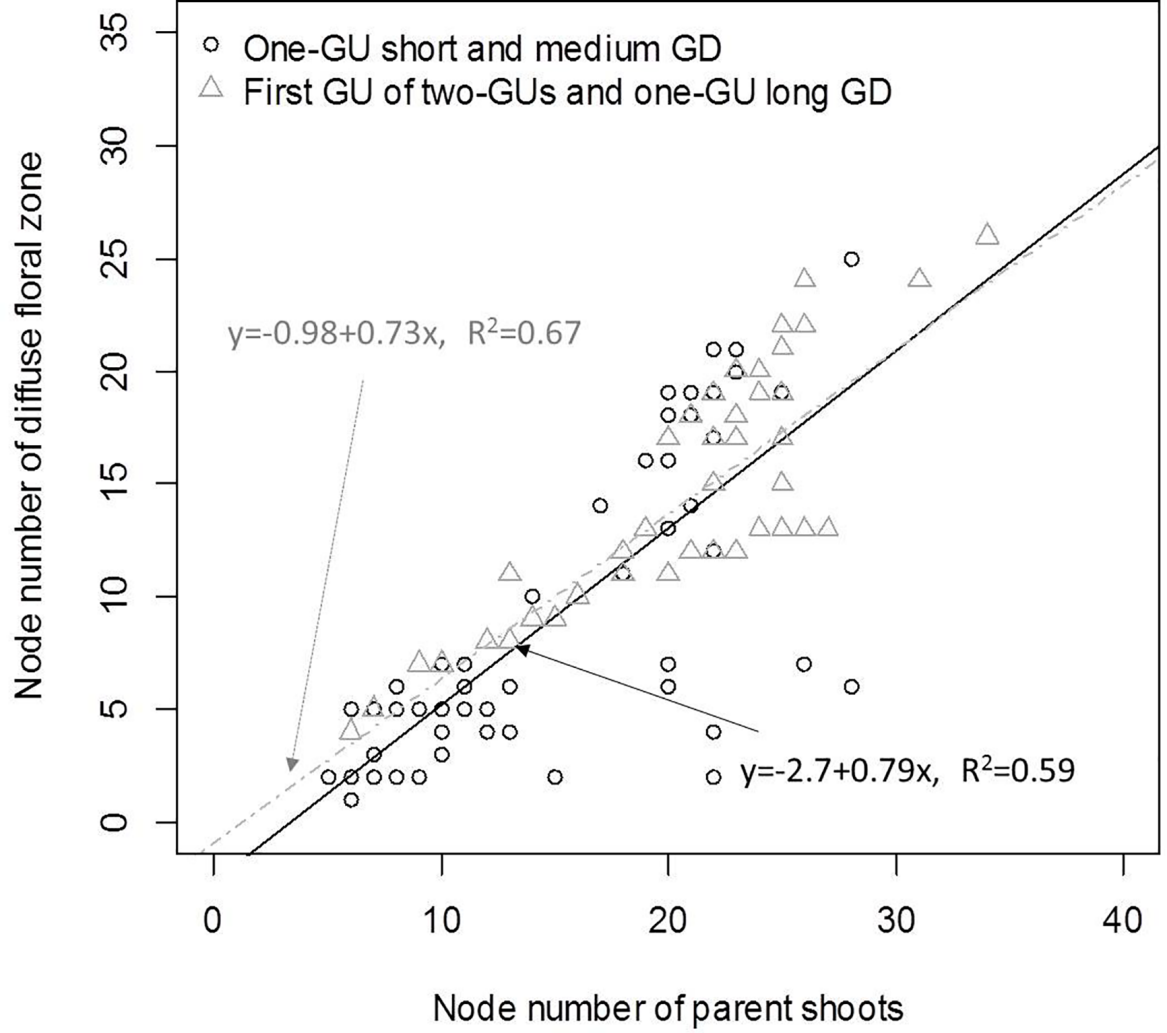
Linear correlation between the length of the diffuse floral zone (state S1 in the model for one-GU with short and medium GD) and the total shoot length of the one-GU with either short or medium GD shoots, and the length of the diffuse floral zone (state L1 in the model for one-GU long GD and two-GUs) and the length of first GU (L0 and L1) for two-GU and one-GU long GD shoots. Here the lengths are the number of nodes. The correlation coefficients (R^2^) were calculated only for the shoots with a diffuse floral zone.

Since the number of nodes per zone and per GU both varied with GD, we investigated their relationship to identify which zone had the greatest effect on the variation of GU length. There was a positive correlation (R² = 0.67) between the number of nodes in the diffuse floral zone (L1) and the total number of nodes for the first GU of the two-GU shoots and the one-GU shoots with a long GD (**Figure 7**). For the one-GU with a short and medium GD, there was a great variability in the length of the diffuse floral zone when the sequences were longer than 20 nodes, which led to a moderate correlation with node number (R² = 0.59). There were no correlations between other zones and the length of the one-GU shoots, the first GU and second GU of the two-GU shoots.

#### Effect of the Growth Period on Branching Patterns

The growth period (GP) affected the occurrence and length of the diffuse floral zone (S1, **Table 2**; **Figure 5**). For the one-GU shoots with either short or medium GD, the diffuse floral zone (S1) occurred slightly more frequently for the shoots with an early GP than with a late GP, while the vegetative zones (S2) were more frequent for the shoots with a late GP than an early GP (**Table 2**; **Figure 5**). For the one-GU shoots with a medium GD, there was a strong impact of GP on the mean length of the diffuse floral zone, as this zone was about three times longer for the shoots with an early GP than those with a late GP, 14.5 vs. 4.8 nodes (**Table 3**; **Figure 5**). There was no difference in the lateral-type distribution for any zone between the different GPs for the one-GU shoots with either a short or medium GD (data not shown). No analysis of GP effect was performed on the one-GU shoots with a long GD because all these shoots in our experiment had an early GP.

For the two-GU shoots, the GP effect on the branching pattern was analyzed for the first GU with short GD and the second GU with long GD because they were the only categories that had both an early and a late GP in our sample. The first GU with short GD had a slightly lower probability of occurrence of the diffuse floral zone (L1) for the early GP than for the late GP (**Table 2**; **Figure 5**, D2). No impact of the GP on zone length was detected for the first GU with short GD (**Table 3**; **Figure 5**, D2). In contrast, the second GU with a long GD had a slightly higher probability of occurrence of the floral zone (L5) and a much lower probability of occurrence of the short shoot zone (L4) when it developed during the early GP compared to the late GP. Also, this GU category had more latent buds in the short shoot zone (L4), and a higher proportion of long laterals (bourse or vegetative shoots) in the floral (L5) and vegetative (L6) zones when it developed during the early GP compared to the late GP (**Figure 6D**).

## Discussion

The branching patterns of different shoot categories of apple cv. “Fuji” were modeled using hidden semi-Markov chains with three transient states for the one-GU shoots with short and medium GD, i.e., monocyclic shoots, and seven transient states for the one-GU shoots with long GD, i.e., monocyclic shoots, and the two-GU shoots, i.e., bicyclic shoots. This strategy was successful because the branching characteristics were homogeneous within a zone but varied between successive zones similar to what has previously been found in apple (Costes and Guédon, 1997; Costes and Guédon, 2002; Renton et al., 2006). Here, we examined the shoot growth rhythm, duration, and period to understand their respective roles on the branching pattern for the following year.

Three zones were common and they were located in the same relative positions independent of the shoot category. They were, therefore, identified in both models: all shoots started with a basal latent zone followed by a diffuse floral zone and ended with a vegetative zone. The existence of a basal latent zone was observed for all shoot categories and more generally at the base of all GUs. Interestingly, such a zone has been found for many species that have been studied so far in the Rosaceae family (Costes et al., 2014) and other perennial species (Meier et al., 2012). The lateral buds that remained latent at the shoot base or at the beginning of the second GU likely result from a strong inhibition by apical control (Bangerth, 1994; Cline, 1997). This apical dominance could result in a very low organogenetic activity during the year of parent shoot growth, thus limiting their outgrowth capacity for the following year. They constitute a bud bank that allows perennial plants to react to pruning and to regenerate epicormic shoots following damage and during aging (Gordon et al., 2006; Gordon and Dejong, 2007; Meier et al., 2012).

The second zone present in all shoot categories is the floral diffuse zone (S1 and L1). In monocyclic shoots with short or medium GD, this zone, i.e., S1, was diffuse and characterized by a low proportion of floral laterals. In bicyclic shoots, a similar zone with a low density of floral laterals, i.e., L1, was found before the growth cessation. The low density of flowers may result from competition for resources. Indeed, the bud fate in this zone was probably determined during the period of new shoot growth after summer pruning. Similarly to the previous study by Lauri and Terouanne (1998), the co-occurrence of the growth of the second GU and the initiation of lateral buds may strongly compete for resources, with the superior strength of demand for apical development of the second GU leading to a reduced floral induction for the laterals of the first GUs. The diffuse floral zone was the most variable zone in terms of the number of nodes and the most associated with total shoot length and GD.

The presence of a distal vegetative zone (S2 and L6) was also observed in all shoot categories. This zone contained long laterals, and thus corresponded to an acrotonic zone. This zone was mainly observed in monocyclic shoots with long GD and bicyclic shoots. For these shoots, the long laterals near the distal end of the parent shoot could be included both in the floral (L5) and the distal vegetative zones (L6), since numerous floral laterals developed a long bourse shoot. The acrotonic zone is commonly observed in apple shoots (Crabbé, 1984; Cook et al., 1998) and many tree species (Hallé et al., 1978; Barthélémy and Caraglio, 2007). In contrast, the distal vegetative zone (S2) contains a lower number of long laterals and was less frequently found in the monocyclic shoots with a short GD than with a medium GD (**Figure 6A**). The fate of axillary buds could be partly determined before the winter period by the number of primordia included in the winter bud (Costes, 2003). The ability of meristems to grow after the expansion of preformed organs in the winter buds that is a post-dormancy event (Champagnat, 1978; Cook et al., 1998), could be affected by the initial number of primordia in the winter bud (Costes, 2003). This ability appears also modulated by the parent shoot’s GD and GP. We found that the proportion of long laterals developed along the parent shoots increased with the GD (**Table 1**), and, therefore, with their number of nodes, which may in turn define the length of the lateral shoots developed in the next year. This relationship between the length or the number of nodes of the parent shoot and its laterals is part of tree ontogeny (Costes et al., 2003; Barthélémy and Caraglio, 2007). In addition, our results suggest that a late GP could be unfavorable to future lateral shoot growth because the number of long laterals, either floral laterals with long bourse shoots in L5 or vegetative laterals in L6, were reduced when the second GU of bicyclic shoots developed later (**Figure 6D**). This reduction could result from a shorter time before dormancy establishment that may lead to a reduction in the number of preformed organs in the winter bud and, therefore, may hamper future neoformed growth in the next season.

Two additional zones were observed for the shoots with long GD. The first is the short shoot zone (L4) present in the monocyclic shoots with long GD and the bicyclic shoots only. Its presence led to different branching structures of these shoots compared to the monocyclic shoots with short and medium GD. This finding can be interpreted as the result of a higher number of vegetative buds along the parent shoot when the GD increases, and strong competition in the spring after bud burst. It is likely that the high number of buds with the greatest growth potential along monocyclic long GD and bicyclic shoots create a competition for nutrients, including carbon and water, that could lead to the rapid growth cessation of a large proportion of meristems after budburst, thus giving birth to short shoots. As primigenic dominance, i.e., the first bud to burst, may lead to acrotony in apple under cold conditions (Maguylo et al., 2012), the shoots that remain short are usually located below the acrotonic zone, either mixed in a single zone with long and floral laterals when the parent shoot had a short or medium GD or in a specific zone when the GD was long.

The final zone is the second floral zone (L5) that is present below the acrotonic zone in the monocyclic shoots with long GD and the second GU of the bicyclic shoots. The location of this floral zone close to the shoot tip indicates that floral induction in axillary meristems can occur late during the season, more than 60 days after full bloom (DAFB), which is the period of floral transition in the terminal meristems of short shoots (Foster et al., 2003; Hanke et al., 2007). A similar location of a floral zone below the distal end and the acrotonic zone of the parent shoot has been found in peach trees (Fournier et al., 1998; Lopez et al., 2008) and other studies of apple trees (Costes and Guédon, 2002; Renton et al., 2006). Compared to S1 and L1, this zone is denser with floral laterals with up to 90% of axillary buds induced to flower. However, considering the relatively short length of this floral zone, i.e., approximately three nodes, the period during which the axillary buds can be initiated appears limited in time.

A strong pattern of floral laterals was observed along the parent shoots, with two distinct zones, one diffuse, S1 or L1, and one dense, L5. It must be emphasized that the first floral lateral present along parent shoots can always be observed after six to ten nodes from a GU base, corresponding to S0 or L0 mean length. This suggests that the period of floral induction in axillary meristems starts rapidly after bud burst. After a summer growth cessation, a similar number of nodes are located below the first occurrence of floral laterals, i.e., adding L3 and L4 mean length. If we consider the positions of floral zones from the distal end of the parent shoots, i.e., in S1, L1 or L5, the floral induction in axillary meristems always occurred below a growth cessation, whatever the GD and GP. Previous studies also found that the termination of shoot growth is a prerequisite for flower initiation (Luckwill and Silva, 1979; Dencker and Hansen, 1994; Koutinas et al., 2010) and that the decrease of plastochrone rate may favor floral bud initiation (Crabbé, 1984). These statements lead us to interpret floral induction in axillary meristems as resulting from an intermediate organogenetic state. These meristems are not strongly inhibited as this would lead to latency, and they do not grow immediately, as this would lead to syllepsis. However, they must be able to maintain intra-bud growth for developing bourse leaf primordia and floral primordia before entering into dormancy. Based on Heide and Prestrud (2005), growth cessations during fall are brought on by low temperatures and not a decrease in day length. However, other environmental factors such as high temperature, high vapor-pressure deficit, or low soil water availability are likely to affect summer growth cessation. This suggests that the floral induction in an axillary meristem could be triggered by a combination of factors: either its location relative to the apex or the time duration during which the axillary buds stay in the proximity of an apex with organogenetic activity. These two conditions could define the axillary meristem’s ability to maintain intra-bud growth and to develop the minimum number of primordia before floral transition (Koutinas et al., 2010). In addition, fruit load also plays a key role in floral induction. Indeed, under high crop load condition, inhibiting signal coming from fruits restrains floral induction (Dennis and Neilsen, 1999; Belhassine et al., 2019).

## Conclusion

This study showed the similarities and dissimilarities of the branching organization among shoots with different growth characteristics, which were defined by their polycyclism, GD, and GP. The complexity of branching structure increased with GD, with a higher probability of developing floral and vegetative zones and enhanced acrotony. The impact of growth cessation was relatively limited since the shoots that were able to develop a second GU had a branching structure highly similar to those without growth cessation. An extended GD also extended the diffuse floral zone regardless of the further development of the parent shoot. The statistical models used in this study enabled us to provide a precise description of the embedded branching structures, which are likely to help formulate better strategies for tree management, in particular for managing the number of floral laterals desired (or not) in relation to the shoot growth of the parent.

## Data Availability Statement

The raw data supporting the conclusions of this article will be made available by the authors, without undue reservation.

## Author ContributionS

Y-TW devised the study and collected the data, with close supervision from GH and MS-G. Y-TW, EC, and BP analyzed the data. Y-TW, BP, and EC drafted the manuscript. All authors contributed to the article and approved the submitted version.

## Funding

This research was partially supported by Washington State University’s AgWeatherNet Program.

## Conflict of Interest

The authors declare that the research was conducted in the absence of any commercial or financial relationships that could be construed as a potential conflict of interest.
